# A business case for diagnostic stewardship: a practical guide from the Society for Healthcare Epidemiology of America (SHEA) Diagnostic Stewardship Special Interest Group

**DOI:** 10.1017/ash.2026.10839

**Published:** 2026-09-09

**Authors:** Sarah E. Turbett, Florian Daragiati, Victoria T. Chu, Valeria Fabre, Tsun Sheng N. Ku, Jonathan Ryder, Anna Sick-Samuels, Francine Touzard-Romo, Jessica Zering, Daniel J. Morgan, Mohamad G. Fakih

**Affiliations:** 1 Massachusetts General Hospital, USA; 2 Ascension Health, USA; 3 University of California San Francisco, USA; 4 Johns Hopkins School of Medicine: The Johns Hopkins University School of Medicine, USA; 5 University of Montana School of Osteopathic Medicine, USA; 6 University of Nebraska Medical Center College of Medicine, USA; 7 Warren Alpert Medical School of Brown University: Brown University Warren Alpert, USA; 8 Washington State Department of Health, USA; 9 University of Maryland School of Medicine, USA; 10 Wayne State University School of Medicine, USA

## Abstract

**Objective:**

We examined five common targets for diagnostic stewardship that have been associated with improved patient outcomes. For each, we estimated the financial and reputational impact of each intervention on a typical 200 bed community hospital. These estimates can be adjusted for individual hospital estimates.

**Results:**

We found consistent savings and potential improvements in hospital metrics associated with these different diagnostic stewardship interventions. *Clostridioides difficile* (CDI) diagnostic stewardship showed ＄125,617 expected savings plus a likely additional 75% reduction in the hospital-acquired condition (HAC) CDI metric. Urine culture stewardship showed ＄34,111–＄92,361 in savings with an additional 30% reduction in the HAC catheter-associated urinary tract infection metric. Procalcitonin de-implementation showed ＄78,938 expected savings from test discontinuation. Expanded respiratory pathogen panel stewardship showed ＄383,650–＄668,000 in savings, depending on degree of reduction and replacement with focused viral testing. Blood culture stewardship showed ＄1,860,800 in savings with an additional 33%–80% reduction in HAC central line associated bloodstream infection and MRSA bacteremia metrics.

**Conclusions:**

In total, a 200-bed hospital implementing all five interventions could expect an approximate ＄2,482,616–2,825,216 in laboratory and clinical savings. These interventions would also improve many HAC metrics, providing an additional ＄200,000–＄600,000 in savings, depending on baseline performance. Finally, reputation metrics, such as Leapfrog grades, would also improve.

## Introduction

Diagnostic stewardship is an evidence-based framework designed to ensure the right test is ordered for the right patient, at the right time, in the most appropriate care setting, with results reported and interpreted in ways to encourage the right action. Misuse or overuse of diagnostic tests, tests performed in the incorrect patient care setting, inappropriate repeat or serial testing, and unnecessary laboratory test send-outs all contribute to escalating healthcare costs and inefficient care. These practices can also extend hospital length-of-stay (LOS), drive inappropriate antimicrobial use, inadvertently identify surveillance-based, not clinically relevant, healthcare-associated infections (HAIs), and promote the development of antimicrobial resistance. Diagnostic stewardship enhances the chances of achieving the right diagnosis which can improve antimicrobial use and reduce antimicrobial resistance, resulting in better patient outcomes, more efficient care, and improved institutional costs and metrics.^1^

In the United States, diagnostic testing can also impact the hospital-acquired condition (HAC) Reduction Program (in part through higher Laboratory-Identified events [Lab-ID] from unnecessary testing), the Center for Medicare and Medicaid Services (CMS) Star Rating Program, and the Leapfrog Safety Grade Program. Hospitals that perform poorly in these programs can face consequences, including a 1% reduction in Medicare payments for HAC penalties, and reputational consequences from low CMS Star ratings or poor Leapfrog grades.

Recognizing the value of stewardship interventions, the Centers for Disease Control and Prevention (CDC) released its “Core Elements of Hospital Diagnostic Excellence” in 2024, describing key steps needed to improve diagnoses in the hospital setting.^2^ Essential to this recommendation was the application of diagnostic stewardship to commonly misused microbiologic tests such as *Clostridioides difficile,* urine cultures, blood cultures, and multiplex molecular testing panels. A business case analysis for key stakeholders helps support hospital adoption. Prior work has demonstrated the importance of developing business cases for infection prevention and antimicrobial stewardship, showing that a clear and objective financial rationale can enhance program support.^3^

The goal of this article is to provide a simplified framework for business-case development for diagnostic stewardship programs within U.S. healthcare systems. This article addresses key economic measurements related to the diagnostic pathway with an emphasis on the financial impact of a stewardship intervention. To calculate costs, practical measurable outcomes are employed using estimates available from public databases, published literature, or institutional data. Key performance metrics are defined with the goal of improving healthcare quality, safety, and efficiency. Finally, concise business cases for five high-yield diagnostic test stewardship interventions are provided.

## Methodology

### Framework for business-case development

#### Defining the scope and objectives

The first step in business-case development of a medical center or health care systems diagnostic stewardship program is to define the scope and objectives. The scope clarifies the recommended tests or interventions of focus, which are chosen based on alignment with institutional priorities, national safety goals, clinical outcomes, and operational feasibility. In general, it is important to guide decisions about high-value, high-impact interventions with diagnostic utilization data. Common high-yield, low effort targets include respiratory molecular multiplex panels, advanced molecular diagnostics (broad-range polymerase chain reaction, metagenomic next-generation sequencing, microbial cell-free DNA testing), procalcitonin levels, blood, respiratory, and urine cultures, and *C. difficile* testing. Included within the scope for each test or intervention of focus are the following:

- Clear definition of the problem.
- Description of the current state of the diagnostic testing or intervention undergoing evaluation, including an annual cost analysis.
- Outline of the patient care setting(s) where the diagnostic stewardship intervention will occur (e.g., ambulatory office setting, emergency department, inpatient intensive and non-intensive care units).
- Identification of key stakeholders for the proposed stewardship intervention.

The objectives should clearly state the goal of the stewardship intervention. In general, these goals are measurable and clinically meaningful, anchoring on avoiding tests that do not bring clinical value to achieve the proper diagnosis, in concordance with national guidelines. While a cardinal value is enhancing clinical outcomes, with associated reductions in unnecessary antimicrobial days of therapy or reducing hospital LOS, additional focus should be on the expected financial benefits and reputational advantages of the intervention with reference to the HAC Reduction Program, value-based purchasing (VBP), and CMS Star Ratings.^4–6^

#### Calculating the costs of the current state of the diagnostic test targeted for intervention

As one of the core principles of diagnostic stewardship programs is to target inappropriate test use, it is necessary to first calculate the cost of the current state of a diagnostic test targeted for stewardship intervention. By having a “baseline” cost estimate, the total net return on investment (ROI) of the proposed intervention can be calculated. We provide these estimates for five specific cases, but further estimates can be made with the following approach used in this report. Baseline costs can be broken into the following categories, where applicable:

- Laboratory costs: test cost approximation includes reagents and consumables, instrumentation, and overhead needed to perform the testing in current state. This information can be obtained by hospital laboratory leadership as it is calculated routinely in annual budget analyses.
- Information technology (IT)/electronic health records (EHR) costs: it can be assumed that the IT/EHR costs for a specific test are small in comparison to the total IT/EHR related activities of a given institution and are fixed in nature. To account for this, baseline IT/EHR costs can be assumed to be ＄0.
- Clinical costs: clinical costs will vary depending on the test targeted for intervention and can include costs associated with antimicrobial use, isolation precautions, or test-related adverse events as well as current penalties or value-based payments. The impact on hospital LOS can be considered.

Antimicrobial costs can be calculated as days of antimicrobial therapy or cost per dose using cost estimates obtained from pharmacy acquisition costs or 340B ceiling pricing (if applicable).^7,8^ If those are unavailable, average wholesale acquisition costs can be used as rough approximations. Imaging costs can be calculated using internal data from hospital accounting systems^9^ (if available), Medicare cost reports,^10^ or from published literature. Isolation and test-related adverse event costs can be approximated using published literature.

#### Calculating the costs of diagnostic stewardship intervention

Diagnostic stewardship programs require investment at multiple levels. Program investments include project management support, informatics resources for EHR clinical decision support (CDS), and analytics infrastructure for tracking test utilization and outcomes. Clinical investments include physician/provider, pharmacist, and laboratory support for guideline development and education. Finally, laboratory costs may include new test platform acquisition and instrument verification or validation with additional potential longstanding effects on operational budgets depending on reagent and consumable costs, and any additional overhead costs related to sample processing.

Despite the multi-level investment needed to support diagnostic stewardship programs, it is important to note that most hospitals already have the infrastructure in place to easily develop such initiatives. Since the release of CDC’s Core Elements of Hospital Antibiotic Stewardship Programs (ASP) in 2014,^11^ which included formal financial, information technology, and human resources support as a core element of a dedicated antimicrobial stewardship program, and the subsequent Center for Medicare and Medicaid Services (CMS) final rule in 2019 mandating formal ASPs,^12^ most hospitals have implemented formal ASPs, with nearly 95% of acute care hospitals meeting all seven of CDC’s hospital core elements in 2021.^13^ Although additional investments would be needed to expand these programs to include more robust diagnostic stewardship initiatives, the framework for such programs is likely already in place at most centers, mitigating many of the upfront implementation costs. In some settings, some of these costs will even be fixed, allowing for rapid adoption.

Cost savings should incorporate three categories of return:

1. Direct savings from reduced unnecessary testing
  - Calculated as expected number of tests reduced, multiplied by cost per test.
    - Test volume reduction calculation can be based on internal data analysis (compared to hospital baseline) or from proportion of overused tests from the published literature.
    - Test cost can be obtained by laboratory leadership as there is likely some variation in price based on vendor, location, and test volume.
2. Downstream cost avoidance
  - Calculated through reduction in antimicrobial and imaging costs, time in isolation precautions, and test-related adverse events.
    - Antimicrobial costs can be calculated as days of antimicrobial therapy or cost per dose avoided using cost estimates as described above.
    - Imaging, isolation, and test related adverse event costs can be approximated as described above.
3. Reduced penalty-based reductions (HAC Reduction Program) and incentive-based payments (VBP)
  - HAC reduction program and VBP performance are multivariate and influenced by numerous clinical, operational, and environmental factors.
  - Including financial savings from diagnostic stewardship introduces several limitations: the savings are difficult to attribute, challenging to predict, highly dependent on external benchmarks, and often categorized as soft or avoided costs rather than direct savings (revenue generating) compared to the current state VBP adjustment.

Modeling should include conservative and best-case estimates to enhance credibility.

#### Calculating ROI using expected savings

Once the cost and anticipated savings of the diagnostic stewardship intervention are completed, ROI can be calculated using expected savings as a simplified proxy. As hospital budgets operate on an annual cycle, analysis should prioritize demonstrating financial return within the next fiscal year. However, longer time frames–two to three years–should be considered for initiatives requiring capital investments, such as new testing platforms, where a longer ROI is expected.

- Expected savings: Calculated by subtracting future state annual costs (with incorporated savings) from current state annual costs.

#### Ongoing evaluation of key performance metrics

Measures of success need to be defined prior to implementation and monitored prospectively after the diagnostic stewardship intervention has been put in place. Utilization metrics include test volume per patient-days or encounters. Secondary downstream measures include antimicrobial days of therapy, LOS, and publicly reported HAI incidence.^14^ Financial outcomes should capture test cost avoidance, operating margin improvement, penalty dollars avoided, and incentive dollars earned. External outcomes may include performance in CMS Star Ratings and Leapfrog Safety Grades, which have both financial and reputational implications.^4,6,15^

### Business case examples

Below are examples of five diagnostic stewardship targets that are often inappropriately used in healthcare settings. Each test chosen was vetted by subject matter experts (SMEs) in diagnostic stewardship through the Society for Healthcare Epidemiology (SHEA) Diagnostic Stewardship Interest Group and was based on literature review supporting diagnostic stewardship strategies to improve clinical care and reduce unnecessary costs.^15–20^ The impact magnitude, implementation feasibility, and resource intensity were also considered when curating this list, and various strategies were provided, when available, for local adoption.

To design these business case examples, certain assumptions were made by the committee to facilitate development. First, all cost analyses were estimated for a typical 200-bed community hospital with 20 intensive care unit beds. Cost assessments for other types of healthcare facilities such as critical access hospitals, large academic medical centers, or hospitals that serve specific populations (eg, oncology) would need to be adjusted. Second, for each diagnostic test, a “standard practice” scenario was defined to generate a baseline annual cost for each test prior to the stewardship intervention. Each “current state” represents a common real-world example for each test’s stewardship practices in U.S. healthcare facilities; all potential testing options were not modeled. The “standard practice” scenarios for the five business case examples include:

1. *C. difficile* testing: One step testing process (PCR) available and ordered at clinician discretion.
2. Urinalysis/urine culture (UA/Ucx): UA and Ucx ordered at clinician discretion as separate orderables (no link between UA result and Ucx performance).
3. Procalcitonin (PCT): test available in-house and is readily orderable by clinicians; no stewardship in place.
4. Multiplex respiratory pathogen panels: Available in the outpatient (OP)/emergency department (ED)/inpatient (IP) setting and ordered at clinician discretion; no stewardship in place.
5. Blood cultures (Bldcx): Test ordered at clinician discretion, no stewardship or CDS tool (CDST) available.

For each business case example, the scope, objectives, description of the intervention, current state cost analysis, future state cost and savings analysis, and expected savings are described using the framework established above.

### *Clostridioides difficile* testing

#### Diagnostic challenges in *Clostridioides difficile* infection (CDI)

*Clostridioides difficile* infection (CDI) is associated with significant morbidity, mortality, and healthcare costs. Primary CDI increases healthcare expenditures by approximately ＄24,000 per patient, with even higher costs for recurrent infection.^21,22^ Hospitals are also subject to penalties under the Medicare HAC Reduction Program, where hospital-onset CDI (HO-CDI) accounts for 16.7% of the performance score.^23^

CDI is a clinical diagnosis supported by laboratory testing, but hospitalized patients may experience diarrhea for many reasons and frequently have asymptomatic *C. difficile* colonization.^24,25^ Highly sensitive assays such as nucleic acid amplification tests (NAAT) and glutamate dehydrogenase (GDH) immunoassays cannot distinguish colonization from active infection, leading to overdiagnosis if used alone. Toxin immunoassays are more specific but less sensitive and may miss true infections. Current guidelines therefore recommend a two-step algorithm using NAAT and/or GDH screening followed by toxin A/B immunoassay testing.^26^ Implementation of two-step testing has been associated with an approximate 50% reduction in HO-CDI and a 40% reduction in CDI-specific antibiotic use.^27,28^

#### Diagnostic stewardship interventions for CDI

The two primary diagnostic stewardship interventions for CDI are the two-step testing described above, and clinical decision support (CDS) to restrict repeat testing and testing of patients on laxatives (unless the laboratory or infection prevention is contacted for permission), with both interventions likely being additive in impact (see Supplementary Materials (SM) Table 1). For example, CDS combined with testing restrictions achieved a 33% reduction in test ordering and a 57% reduction in HO-CDI rates at a single academic center.^29^ On a larger scale, the Henry Ford Health system reported net savings of nearly ＄7 million across five hospitals, roughly ＄1.4 million per hospital, with two-step testing and hard stop rules limiting repeat testing and testing in the setting of recent laxative use.^30^

#### Return on investment

The ROI for *C. difficile* diagnostic stewardship is driven by reduced unnecessary testing, less antibiotic use for false positive tests, fewer Lab-ID events, and avoided delays in hospital discharge. Implementing a two-step algorithm combined with CDS can feasibly reduce *C. difficile* testing by 33%, and Lab-ID events by 75%, making it a cost-effective strategy (see SM Table 1).^18,27,29,31,32^

### Urine culturing

#### Inappropriate urine culture use in patients with asymptomatic bacteriuria

Asymptomatic bacteriuria is a common condition that does not merit antibiotic treatment outside of select indications (pregnancy, urologic procedures with potential for mucosal injury).^33^ However, inappropriate urine culture testing in patients without urinary tract infection (UTI) symptoms is frequently performed by providers, resulting in unnecessary antibiotic treatment, adverse events, and increased healthcare costs.^34^ Additionally, inappropriate urine culture testing may impact the number of reportable catheter-associated urinary tract infections (CAUTIs) by healthcare facilities as the urine culture result is a key component of the National Healthcare Safety Network (NHSN) definition. This contributes to the HAC score of hospitals that participate in the CMS value-based purchasing program which affects CMS payments.^35^

#### Diagnostic stewardship interventions to prevent unnecessary urine cultures

When evaluating a patient for a UTI, conditional urine culturing is when a urine culture is only performed if predefined UA criteria indicating inflammation are met such as pyuria. Cutoffs are typically < 10 WBC per high power field although different parameters have been suggested and used in clinical practice.^36,37^ Select patient groups are typically excluded from conditional cultures (infants, pregnant women, urology preop patients) to avoid complications or consequences from asymptomatic bacteriuria or missed infection. Conditional urine culturing reduces the number of urine cultures performed, which prevents unnecessary antibiotic use, decreases CAUTI rates, and improves healthcare costs.^38–40^ For example, conditional culturing protocols are associated with a 40% reduction in cultures performed,^19,41–43^ a 30% reduction in CAUTI, and similar reductions in antibiotic use.^41,44^ When combined with CDS requiring testing indication, these outcomes improve even more dramatically with a 30 to 85% reduction in urine culture volume and additional reduction in antibiotic use^38^ (see SM Table 2).

#### Return on investment

The ROI for conditional urine culture with or without CDS indications is driven by cost savings from fewer urine cultures, decreased antibiotic use, and fewer adverse events, including CDI. Approximate cost savings with these interventions have been estimated to exceed ＄1,800,000 over 6 hospitals (based on a 21% urine culture reduction and a cost per urine culture of ＄39), or more than ＄200,000 per year for a 200-bed hospital^39,40^ (see SM Table 2).

### Procalcitonin

#### Inappropriate use of procalcitonin

Although procalcitonin (PCT) has been shown to be a potentially useful biomarker for reducing antibiotic treatment durations in pneumonia^45^ and sepsis,^46^ clinical studies have consistently demonstrated a limited real-world impact, likely due to high reliance on adherence to PCT-guided antibiotic treatment algorithms for success.^47,48^ Real-world PCT implementation in adult populations has been plagued with improper use and low adherence to defined algorithms.^49–52^ As a result, various diagnostic stewardship strategies have been attempted to improve adherence, including test order restriction^53^ and electronic medical record CDSTs,^54,55^ with limited success. In addition, in recent years, the recommended antibiotic therapy durations for community-acquired pneumonia have been reduced to as few as 3–5 days,^56–58^ further decreasing the value of PCT in clinical practice. Thus, multiple institutions have chosen to forego PCT entirely.

#### Diagnostic stewardship interventions for procalcitonin

Recognizing the above problems with PCT and its high cost, it is an optimal target for de-implementation. A baseline assumption of low adherence to PCT-guided antibiotic treatment algorithm is made with the intervention of discontinuing PCT use in the adult population (see SM Table 3). As PCT is recommended for use in febrile infants,^59^ this population is excluded from this exercise.

#### Return on investment

The ROI for PCT de-implementation is relatively simple with cost savings driven by reduction in PCT test performance (see SM Table 3). As the baseline assumption is one of limited to no adherence to a PCT-guided treatment algorithm, PCT discontinuation is modeled to have no effect on antibiotic use or antibiotic associated adverse events.

### Expanded multiplex respiratory pathogen panels

#### Limited clinical utility of expanded respiratory pathogen panels (RPPs)

Use of RPPs has been broadly adopted over the past decade and saw a sharp rise in use after the SARS-CoV-2 pandemic.^60,61^ However, widespread implementation has not demonstrated improvement in clinical outcomes including antibiotic prescribing, emergency department (ED) length of stay, or hospitalization. The only impact identified has been the appropriate use of influenza antivirals.^62–65^ In contrast, economic analysis of targeted first line testing with a narrow panel for clinically high-yield viruses such as influenza, respiratory syncytial virus (RSV), and SARS-CoV-2 can improve healthcare resource utilization^66^ with the same impact on appropriate rapid antiviral initiation.^67,68^ In addition, Medicare coverage policy differentiates between smaller (≤5 targets) and larger (>5 targets) multiplex panels in outpatient or ED-only settings^68^; testing panels with fewer targets (eg, influenza A/B, RSV, and SARS-CoV-2 only) align better with reimbursement policy and clinical utility, especially when rapid results can actively inform clinical management decisions (eg, whether to prescribe an antiviral). Due to limited evidence showing improved health outcomes, expanded RPPs (panels targeting additional respiratory pathogens plus influenza A/B, RSV, and SARS-CoV-2) are generally not considered medically necessary for patients with self-limited illnesses and can result in reduced or denied payor coverage unless specific criteria are met.^69^ Consequently, extended RPP testing may lead to significant non-reimbursed hospital costs or increased out-of-pocket burden for patients, up to ＄1,800 per test.^70^ Consequently, many hospitals have implemented diagnostic stewardship strategies to optimize application of expanded RPP testing and avoid low value patient care and excess costs.^71^

#### Diagnostic stewardship interventions for expanded RPPs

To improve expanded RPP diagnostic stewardship, CDS tool implementation with an order prompting clinicians to perform targeted testing rather than expanded RPP for scenarios in which a limited viral panel is appropriate (eg, patients with mild respiratory symptoms being discharged to home) can be considered (see SM Table 4). Expanded RPPs would then be reserved for higher yield scenarios (immunocompromised patients or those being admitted to the intensive care unit). Such approaches have demonstrated a 33–40%^72^ reduction in expanded RPP testing, reducing costs by preventing overutilization.^72,73^ Healthcare systems with mandatory upfront limited targeted testing algorithms (no upfront expanded RPP testing) have seen even larger (60%) reductions in expanded RPP testing (internal communication from MGF).

#### Return on investment

Practically, the ROI for expanded RPP CDS is driven by their substitution with less costly smaller panel testing (influenza A/B, RSV, and SARS-CoV-2). However, CDS may also reduce overall respiratory test volume by reducing low-yield testing, resulting in further cost savings.^74^ Our model assumes no change in antibiotic prescribing and adverse events with expanded RPP CDS implementation (see SM Table 4).

### Blood cultures

#### Suboptimal blood culture ordering practices

Bloodstream infections are a significant cause of morbidity and mortality, and blood cultures remain the cornerstone of diagnosis.^75^ However, blood cultures are often overused in the clinical setting,^76–80^ and focused stewardship interventions to improve ordering practices is an emergent clinical need. Additionally, blood culture collection and processing are resource intensive; even a modest decrease in blood culture utilization (20%) can result in significant cost savings. Although there is less data on the impact of blood culture stewardship interventions on reducing antibiotic use or the misidentification of central line associated bloodstream infections (CLABSI), some studies have reported a beneficial impact on these outcomes which would further lead to cost reductions and a reduction in CLABSI and MRSA bacteremia by 33–80%.^81,82^ Finally, 10–30% of positive blood cultures represent blood culture contamination, which has been associated with unnecessary antibiotic use, longer hospital stays, unnecessary healthcare resource use, and misdiagnosis of sepsis.^83,84^ Efforts to reduce contamination rates, particularly in patients with low likelihood of bacteremia, not only improve clinical outcomes, but also reduce costs, with reports of up to twice the savings compared to reducing unnecessary blood cultures in that population alone.^85^

#### Diagnostic stewardship interventions targeting blood culture collection and blood culture contamination

Interventions targeting blood culture collection only in scenarios with high probability of bacteremia (sepsis/septic shock, endovascular infection, meningitis, etc..) or for when documentation of bacteremia clearance is needed to guide management (eg, *S. aureus* bacteremia), can reduce blood culture utilization by up to 30% with no impact on mortality or readmission rates (see SM Table 5). Evidence-based strategies to prevent contamination, including chlorhexidine for skin antisepsis and standardized collection by trained phlebotomists, reduce blood culture contamination by 40–60%.^78,86^

#### Return on investment

ROI is largely driven by the cost saved by reducing the overall number of blood culture bottles used and by preventing unnecessary laboratory work up of contaminated cultures. While the costs associated with unnecessary antibiotic use, CLABSI misattribution, and hospital length of stay can also be considered (see SM Table 5).

## Reputational and financial impact of reduction in healthcare-associated infections

A recognized driver for adoption of diagnostic stewardship is its positive impact on healthcare-associated infection surveillance events including *C. difficile* infection (75% reduction), CAUTI (30% reduction), CLABSI and MRSA bacteremia (33–80%).^16^ All metrics are based on laboratory testing. Beyond reputational impact of publicly reported HAIs, these metrics are also part of the CMS HAC reduction program, along with the CMS patient safety and adverse events composite measure and surgical site infections. Hospitals are penalized with a 1% reduction in overall CMS reimbursement if they are in the worst 25% of hospitals nationwide.^87^ For a typical 200-bed community hospital, this 1% reduction is estimated at ＄200,000–＄600,000 annually.^14,88,89^ The impact of adopting the three diagnostic stewardship interventions for *C. difficile,* conditional urine culturing, and blood culture stewardship could significantly reduce 4 of 6 HAC metrics, making hospitals much less likely to have a ＄200,000 to ＄600,000 annual penalty.

## Conclusion

Diagnostic stewardship is a cost-effective approach to improving patient care and optimizing healthcare delivery. Here, we have provided a framework for business-case development for diagnostic stewardship interventions and provide examples of these business cases for five commonly misused diagnostic tests and the potential savings attributed to each (see Table 1). Healthcare facilities should adopt these templates to meet the needs of their institutions with the goal of rapid implementation of these measures to optimize clinical outcomes and operational efficiency.

**Table 1.** Impact of diagnostic stewardship interventions on testing, HAIs and costs Table 1 long description.

	Impact on testing and laboratory costs	Impact on clinical costs	Impact on HAIs	Net annual savings^a^
2-step *C. difficile* testing with clinical decision support	33% decrease	58% decrease	75% decrease	＄125,617
Conditional urine culturing with clinical decision support	40% decrease	27% decrease	30% decrease	＄34,111-＄92,361
Procalcitonin de-implementation	100% decrease	No impact	No impact	＄78,938
Expanded Respiratory pathogen panel stewardship with clinical decision support	30–60% decrease	No impact	No impact	＄383,650-＄668,000
Blood culture clinical decision support and a bundle to reduce blood culture contamination	30% decrease	63% decrease	33–80% impact	＄1,860,300
**Total**	**30–100% decrease**	**27–63%**	**30–80%**	**＄2,482,616–＄2,825,216**

a Net annual savings does not include potential HAI reductions. Details about how net savings were calculated can be found in the supplementary materials.

HAI: healthcare-associated infections.

## Supporting information

10.1017/ash.2026.10839.sm001 Turbett et al. supplementary material Turbett et al. supplementary material

## Supplementary material

The supplementary material for this article can be found at https://doi.org/10.1017/ash.2026.10839.

## Table 1. Long description

The table presents data on the impact of various diagnostic stewardship interventions on testing and laboratory costs, clinical costs, healthcare-associated infections (HAIs), and net annual savings. It has five rows and five columns. The columns are labeled ‘Intervention’, ‘Impact on testing and laboratory costs’, ‘Impact on clinical costs’, ‘Impact on HAIs’, and ‘Net annual savings’. The rows list different interventions and their respective impacts. Row 1: 2-step C. difficile testing with clinical decision support, 33 percent decrease, 58 percent decrease, 75 percent decrease, ＄125,617. Row 2: Conditional urine culturing with clinical decision support, 40 percent decrease, 27 percent decrease, 30 percent decrease, ＄34,111-＄92,361. Row 3: Procalcitonin de-implementation, 100 percent decrease, no impact, no impact, ＄78,938. Row 4: Expanded Respiratory pathogen panel stewardship with clinical decision support, 30-60 percent decrease, no impact, no impact, ＄383,650-＄668,000. Row 5: Blood culture clinical decision support and a bundle to reduce blood culture contamination, 30 percent decrease, 63 percent decrease, 33-80 percent impact, ＄1,860,300. The total impact ranges from 30-100 percent decrease in testing and laboratory costs, 27-63 percent decrease in clinical costs, 30-80 percent decrease in HAIs, and net annual savings of ＄2,482,616-＄2,825,216.

Navigate back to Table 1..
