# Supplementary material for: A business case for diagnostic stewardship: a practical guide from the Society for Healthcare Epidemiology of America (SHEA) Diagnostic Stewardship Special Interest Group
Source: Antimicrob Steward Healthc Epidemiol. 2026 Sep 9;6(1):e266. doi: 10.1017/ash.2026.10839 (PMC13760406; doi:10.1017/ash.2026.10839)
Supplement: Supplementary file 1 [file S2732494X26108390sup001.docx]

Supplementary materials:

**Example diagnostic stewardship business cases**

Contents

[Table 1. Business case example for 2-step *Clostridioides difficile* testing with clinical decision support (CDS)	2](#_Toc228875084)

[Table 2. Business case example for conditional urine culturing with clinical decision support (CDS) requiring testing indication	4](#_Toc228875085)

[Table 3. Business case example for procalcitonin de-implementation.	6](#_Toc228875086)

[Table 4. Business case example for a real-time clinical decision support tool (CDS) for expanded respiratory pathogen panel stewardship	7](#_Toc228875087)

[Table 5. Business case example for blood culture clinical decision support and implementation of a bundle to reduce blood culture contamination	9](#_Toc228875088)

[References	11](#_Toc228875089)

# Table 1. Business case example for 2-step *Clostridioides difficile* testing with clinical decision support (CDS)

**Diagnostic stewardship intervention: Clinical decision support combined with a 2-step algorithm for *Clostridioides difficile* testing**

**Setting:** 200 bed, 20 ICU bed community hospital [200 x 0.9 occupancy rate x 365 days = 65,700 Patient Days (PD) per year]

**Baseline State: *C. difficile* NAAT as one step test ordered at discretion of physician, no clinical decision support or test restriction**

Annual cost:

- Laboratory:
  - # tests (20 tests/1,000 PD x 65,700 PD per year = 1,314 tests per year^a^
  - 1-step NAAT: $40/test^b^ x 1314 tests per year **= ~ $52,560 per year**
- IT/EHR maintenance/stewardship oversight: **$0** (Fixed cost to institution)
- Clinical: Assuming a positive rate of 20%^c^ (0.2 x 1,314) = 263 positive tests per year
  - Treatment cost (assuming a 10-day course for each positive case)
  - Oral vancomycin (~ $75 per 10-day course)^d^
    - If 70%^e^ of positive cases get oral vancomycin (263 x 0.70 x $75) = ~**$13, 800 per year**
  - Oral Fidaxomicin (~$2,400 per 10-day course)^f^
    - If 30%^e^ of positive cases get oral fidaxomicin (263 x 0.3 x $2,400) = ~**$189,360 per year**

**Total: $250,720 per year**

**Objective:**

- 33% decrease in CDI test order rate per 1,000 patient days^g^
- 75% decrease in NHSN HO-LabID events^h^ (50% from CDS and 50% of remaining testing undergoing 2-step testing)

**Stewardship Intervention Description:**

- 2-step algorithm (NAAT followed by toxin EIA assay)^i^ with a result nudge for positive NAAT/neg EIA suggesting colonization
- Hard stop CDS tool with indications for CDI testing (at least 3 loose BM in 24h, no laxatives within 48h, no alternative explanation, no CDI tests within 7 days, limit in patients < 2 years old).

**Clinical Impact of Intervention:**

- Reduction in inappropriate CDI testing and reduction in CDI rates (# tests, positive rate, including HO-CDI per NHSN Lab ID events)
- No significant change in inpatient mortality, ICU admission, CDI treatment, and colectomy for megacolon (balance measures)

**Costs after stewardship implementation (future state):**

Future cost:

- Laboratory:
  - # tests for NAAT = 67% of tests after CDS x 1,314^a^ = 880 NAAT tests per year
  - 1^st^ step NAAT = $40^b^/NAAT x 880 = **$35,200 per year**
  - Assuming a NAAT positivity rate of 20%, NAAT^c^ reflex to toxin EIA [(0.2 x 880 x $28/toxin EIA (includes cost of 1 technologist/day)] = **$4,928 per year**
- Clinical
  - Treatment cost (assuming 176 NAAT + cases (20% positive rate), 50% of cases are NAAT+ are EIA+ and all are treated; and 50% of NAAT+ are EIA-, of whom 25% are treated^)j.^ NAAT+/EIA+ = 0.5 x 176 = 88 patients, all treated. NAAT+/EIA- = 88 patients of whom 22 (25%) are treated = 110 total patients treated
    - Oral vancomycin: if 70%^e^ of positive cases treated with oral vancomycin (110 x 0.7 x $75) ^d^ = **$5,775 per year**
    - Oral fidaxomicin: if 30%^e^ of positive cases treated with oral fidaxomicin (110 x 0.3 x $2,400)^f^= **$79,200 per year**

**Total: $125,103 per year**

**Net annual savings: $125,617 ($250,720 baseline-$125,103 future costs)**

^a^ Calculation based on *C. difficile* testing rate of 20 tests per 1,000 patient days. *C. difficile* testing rates vary across hospitals with reports of 7-20 tests per 1,000 patient days^1^.

^b^ *C. difficile* NAAT costs $30–$50 per test; but charges may be higher depending on the setting^2^.

^c^ *C. difficile* NAAT positive rate reported ~15–20% among hospitalized patients in acute care hospitals^1^. Positive rates will vary by institution, and calculations should be individualized.

^d^ Oral vancomycin cost estimated using hospital compounded vancomycin. Cost estimates will differ depending on vancomycin formulation (compounded liquid vs. capsule).

^e^ Oral vancomycin vs. fidaxomicin treatment rates vary by institution and individual treatment rates should be used for calculations when available. If treatment rates are unknown, published literature reports that roughly 70% of patients receive oral vancomycin and 30% receive fidaxomicin^3^.

^f^ Estimated price of generic fidaxomicin based on WebMDRx reports but prices will depend on pharmacy and discounts.

^g^ CDS combined with two-step testing algorithms, CDS alone, and two-step testing alone have been shown to reduce *C. difficile* testing by 20-33%^4,5^.

^h^ CDS alone has been shown to reduce HO-CDI/Lab-ID rates by 31-58%. For purposes of the business case analysis, a 50% reduction in HO-CDI/lab-ID rates was chosen^4^. Two-step algorithms alone have been shown to reduce HO-CDI/Lab-ID event rates by 57-75%^4,6^. For purposes of the business case analysis, a 50% reduction was chosen. When combined, CDS plus two-step testing is expected to result in an overall 75% reduction in HO-CDI/Lab-ID event rates (initial 50% from CDS followed by an additional 50% [25% or remaining testing post CDS] via two-step testing)^5^.

^i^NAAT/toxin was selected for this analysis. Other two-step algorithms include GDH/toxin and GDH/NAAT. Approximately 55-70% of patients undergoing NAAT/EIA testing can have discordant results (NAAT+/EIA-), but 71% still receive treatment^7,8^.

NAAT: Nucleic acid amplification test; IT: information technology; EHR: electronic health record; HO-CDI: hospital onset *Clostridioides difficile* infection; NHSN: national healthcare safety network; GDH: glutamate dehydrogenase; EIA: enzyme immunoassay; CDS: clinical decision support; BM: bowel movement; h:hour; ICU: intensive care unit.

# Table 2. Business case example for conditional urine culturing with clinical decision support (CDS) requiring testing indication

**Diagnostic stewardship intervention: Conditional urine culturing only in patients with pyuria with CDS requiring testing indication**

**Setting:** 200 bed, 20 ICU bed community hospital

**Baseline State: Stand-alone urine culture ordered at provider discretion (i.e., urinalysis available separately but ordered with urine culture as a separate test)**

Annual cost:

- Laboratory: ($31,612 total)
  - # Urine culture tests/year = 3,700^a^
  - # of positive urine cultures = 22%^b^ x 3,700 cultures = 814 positive cultures per year
  - Urine culture cost:
    - Negative urine culture = (3,700-814) x $6.06^c^ = **$17,489 per year**
    - Positive urine culture = 814 x $17.35^d^ = **$14,123 per year**
- Clinical (assuming 80% of positive urine cultures are treated with a 7-day course of ceftriaxone or similar cost antibiotic (~$12.32 per 7-day course^e^)
  - Antibiotic use
    - # treated cases: 0.8 x 814 = 651 patients treated per year
    - Estimated treatment cost = $12.32 x 651 = **$8,020 per year**
  - CDI/adverse events (~0.5-2% of those on antibiotics develop CDI^f^)
    - Total cases: 0.5%-2% x 651 = 3.3-13.2 per year
    - Total cost of CDI: $22,500^g^ x 3.3-13.2 = **$72,250-297,000 per year**

**Total: $111,882-$336,632 per year**

**Objective:**

- 40%^h^ decrease in urine cultures
- 30%^i^ decrease in antibiotic administration
- 30%^i^ reduction in CDI events

**Stewardship Intervention Description:**

- CDS tool requiring order indication for testing (dysuria, urgency, suprapubic tenderness)
- Conditional urine culturing only with pyuria
- Routine urine culture order is only available for specific patients (i.e., pre-urologic procedure with mucosal trauma, pregnancy, infants)

**Clinical Impact of Intervention:**

- Reduction in unnecessary urine cultures and antibiotic use
- Reduction in CDI events
- No significant change in missed UTI’s or blood stream infections from presumed urinary source

**Costs after stewardship implementation (future state):**

Future cost

- Laboratory:
  - # urinalysis tests performed: assume no change compared to baseline state as already routinely ordered with urine culture **(net cost = $0)**
  - 60% of urine cultures performed after reflex = 0.60 x 3,700 = 2,220 per year
  - # positive urine cultures = 0.22^b^ x 2,220 = 488
  - Urine culture cost:
    - Negative urine culture = (2220-488) x $6.06^c^ = **$10,496 per year**
    - Positive urine culture = 488 x $17.35^d^ = **$8,467 per year**
- Clinical (assuming 80% of positive urine cultures will receive a 7-day course of ceftriaxone or similar cost antibiotic[$12.32 per 7-day course])
  - Antibiotic use
    - # treated cases: 0.8 x 488 =390 per year
    - Estimated treatment cost = $12.32^e^ x 390 = **$4,808 per year**
- CDI/adverse events (~0.5-2% of those on antibiotics develop CDI^f^)
  - Total cases: 0.5%-2% x 488 = 2.4-9.8 per year
  - Total cost of CDI: $22,500^g^ x 2.4-9.8 = **$54,000-220,500 per year**

**Total: $77,771-$244,271 per year**

**Net annual savings: $34,111-$92,361 ($111,882-$336,632 per year baseline –$77,771-$244,271 future costs)**

^a^Urine culture test volume based on data from Ascension Healthcare.

^b^ 22% positive urine culture rate chosen based on published reports by Yogo and colleagues^9^. Positive rates may vary by institution, and numbers should be individualized.

^c^ Cost of negative urine culture includes supply and personnel costs and is based on published reports by Yeates and colleagues^10^.

^d^ Cost of positive urine culture based on identification of one organism per culture and includes supply and personnel costs is based on published reports by Yeates and colleagues^10^.

^e^ Cost of 7-day ceftriaxone course based on internal data from Billings Clinic charges; treatment costs may vary by institution.

^f^CDI rates after antibiotic treatment for UTI range from 0.5-7% depending on the population and underlying risk factors^11,12^.

^g^ CDI cost is based on the 2015 Agency for Healthcare Research and Quality (AHRQ) estimate of $9,341–$25,180 and has been adjusted to reflect inflation. Cost does not include HAC penalties^13^.

^h^ Studies have shown anywhere from 20-85%^9,14,15^ reduction in urine cultures with CDS.

^i^30% reduction in antibiotic use and CDI events based on the assumption that 30% of urine cultures would be reduced with the diagnostic stewardship intervention.

ICU: intensive care unit; CDI: *C. difficile* infection; CDS: clinical decision support; UTI: urinary tract infection.

# Table 3. Business case example for procalcitonin de-implementation.

**Diagnostic stewardship intervention: Procalcitonin (PCT) De-implementation**

**Setting:** 200 bed, 20 ICU bed community hospital

**Current State: Onsite PCT testing available with no PCT-guided ordering or treatment algorithm in place. No PCT stewardship or monitoring available**

Baseline annual cost:

- Laboratory:
  - # PCT tests = 2,900 per year^a^
  - PCT test (includes reagents, instrumentation, and overhead cost): $27.22/test^b^ x 2,900 = **$78,938 per year**
  - IT/EHR maintenance/stewardship oversight: **$0** (Fixed cost to institution)
  - Clinical
    - No expected changes to antibiotic duration or antibiotic related adverse events despite PCT availability **(net cost = $0)**

**Total: $78,938 per year**

**Objective:**

- 100% decrease in PCT testing in adult patients

**Stewardship Intervention Description:**

- Collaborate with the clinical laboratory and the antimicrobial stewardship team to de-implement PCT and re-educate clinicians

**Clinical Impact of Intervention:**

- No significant change in antibiotic duration or antibiotic related adverse events

**Costs after stewardship implementation (future state):**

Future cost:

- Laboratory:
  - # PCT tests = **0** per year
  - PCT cost per test: $27.22 x 0 = **$0 per year**
- Clinical
  - No expected changes to antibiotic duration or antibiotic related adverse events despite PCT unavailability **(net cost = $0)**

**Total: $0 per year**

**Savings (Current State-future state):**

Net annual savings**: $78,938 ($78,938 baseline – $0 future costs)**

^a^Annual PCT test volume based on internal data from a 200-bed community hospital provided by Ascension Healthcare.

^b^PCT cost based on data from 2025 update of the Medicare Clinical Laboratory Fee Schedule. Accessed March 8^th^, 2026. Available at <https://www.cms.gov/medicare/payment/fee-schedules/clinical-laboratory-fee-schedule-clfs/files/25clabq4>.

ICU: intensive care unit; IT: information technology; EHR: electronic health record

# Table 4. Business case example for a real-time clinical decision support tool (CDS) for expanded respiratory pathogen panel stewardship

**Diagnostic stewardship intervention: Real-time clinical decision support for appropriate use of multiplex expanded Respiratory Pathogen Panels (RPP) in the Emergency Department (ED)**

**Setting:** 200 bed, 20 ICU bed community hospital

**Current State: Expanded RPP available in the ED setting and ordered at clinician discretion with no diagnostic stewardship program in place**

Baseline annual cost:

- Laboratory:
  - # of RPP tests (33% of ED visits include RPP testing^a^ x 33,711 ED visits a year^b^): **11,125** RPPs per year
  - RPP cost per test: $150^c^ x 11,125 test per year = **$1,668,750** per year
- IT/EHR maintenance/stewardship oversight: **$0** (Fixed cost to institution)
- Clinical:
  - Assuming no change to antibiotic prescribing, duration, or antibiotic associated adverse events despite expanded RPP availability **(net cost = $0)**

**Total: $1,668,750 per year**

**Objective:**

- 30-60%^d^ reduction in expanded RPP use in the ED (replaced with testing for FLU/RSV/SARS-CoV-2)

**Stewardship Intervention Description:**

Expanded RPP CDS tool with a BPA message that requires documentation of testing indication (e.g., immunocompromised hosts, admission to the ICU) and nudges providers to order targeted viral panel testing for FLU, RSV, and SARS-CoV-2 instead

**Clinical Impact of Intervention:**

- Reduce unnecessary diagnostic testing and associated costs
- Decrease ED patient throughput delays caused by waiting for expanded RPP results

**Costs after stewardship implementation (future state):**

Future Cost:

- Laboratory:
  - # Expanded RPP tests: (Assumes 30%-60% reduction in extended RPP orders that are replaced with targeted testing for FLU/RSV/SARS-CoV-2): 0.4 x 11,125 to 0.7 x 11,125 = 4,450 - 7,788 tests per year
  - # FLU/RSV/SARS-CoV-2: 0.3 x 11,125 to 0.6 x 11,125 = 3,338-6,675 per year
  - Expanded RPP cost per test: $150^c^ x 4,450-7,788 = **$667,000 - $1,168,200** **per year**
  - FLU/RSV/SARS-CoV-2 cost per test: $50^c^ x 3,338-6,675 tests = **$116,900-$333,750 per year**
- Clinical:
  - Assuming no change to antibiotic prescribing, duration, or antibiotic associated adverse events despite expanded RPP reduction **(net cost = $0)**

**Total: $1,000,750 - $1,285,100 per year**

**Savings (Current State-future state):**

**Net annual savings: $383,650-$668,000 ($1,668,750 baseline – $1,000,750-1,285,100 future costs)**

^a^Expanded RPP testing volume is based on published data of testing rates and may over-or under-estimate local values^16^.

^b^ Anticipated volume of ED visits is based on public datasets of ED visits by ED size^17^.

^c^ Cost per test may vary widely across settings^18^, and we present a mid-range estimate of cost at $150 per expanded RPP and $50 for Flu/RSV/COVID panel, although expanded RPP costs may range much higher. We have also assumed a direct shift to targeted viral panel rather than no testing at all, which may underestimate savings.

^d^ Based on prior studies demonstrating a 33-40% decrease in testing after implementing such guidance as well as internal data from Ascension Healthcare showing a 60% reduction in testing with CDS^19,20^.

ICU: intensive care Unit; IT/EHR: information technology/electronic health record; CDS: clinical decision support; BPA: best practice alert.

# Table 5. Business case example for blood culture clinical decision support and implementation of a bundle to reduce blood culture contamination

**Diagnostic stewardship intervention:**

**Blood culture clinical decision support tool to reduce low yield blood cultures**

**Evidence-based bundle to reduce blood culture contamination**

**Setting:** 200 bed, 20 ICU bed community hospital

**Current State:** Stand-alone blood cultures ordered at provider discretion (no CDST in place). Inconsistent and incomplete blood culture contamination prevention and reporting measures^a^

Baseline annual cost:

- Laboratory:
  - # blood cultures: 9,000 per year^b^
  - Blood culture cost: $118^c^/test x 9,000 = **$1,062,000 per year**
- IT/EHR maintenance/stewardship oversight: **$0** (Fixed cost to institution)
- Clinical: Assuming a 3% false positive (contamination) rate^d^
  - BCC treatment cost: $9,000^e^ x (0.03 x 9,000) = **$2,430,000 per year**

**Total annual cost: $3,492,000 per year**

**Objective:**

- 30% reduction in blood culture utilization (focusing on elimination of low-yield cultures)^f^
- 50% reduction in blood culture contamination rates^g^

**Stewardship Intervention Description:**

- Implement a blood culture CDST to reduce low yield blood cultures (post-operative fever, surveillance cultures, isolated fever or leukocytosis, cystitis, non-severe community acquired pneumonia)
- Implement an evidence-based bundle to reduce blood culture contamination per ASM recommendations

**Clinical Impact of Intervention:**

- Reduction in inappropriate blood culture use
- Reduction in BCC
- Decrease in antibiotic use and CLABSI rates^h^

**Costs after stewardship implementation (future state):**

Future cost:

- Laboratory:
  - # blood cultures: 70% of blood cultures after CDS x 9,000^b^ = 6,300 per year
  - Blood culture cost: $118^c^/test x 6,300= **$743,400 per year**
- Clinical:
  - Sterile kit implementation to improve BCC rates: $6^i^ x 6,300 = **$37,800 per year**
  - BCC treatment cost: $9,000^e^ x (1.5% contamination rate after BCC bundle x 6,300) = **$850,500 per year**

**Total: $1,631,700 per year**

**Savings (current state-future state)**

**Net annual savings: $1,860,300 ($3,492,000 baseline- $1,631,700 future costs)**

^a^Baseline state based on data reported by Fabre and colleagues^21^.

^b^ Based on internal data from Ascension Healthcare.

^c^Represents an estimated base cost for a single blood culture (includes cost of labor, supplies, instrumentation, and overhead)^22^.

^d^ A target blood culture contamination rate of <3% is used by most hospitals and is considered a reasonable target per the Clinical Laboratory Standards Institute^21,23^.

^e^BCC cost varies from $4,000-$9,000 based on the literature^24^.

^f^Available literature suggests that blood culture CDSTs can reduce blood culture use by 20-30%^23,25^.

^g^Evidence-based methods can reduce BCC from 40-60%^26^.

^h^Blood culture CDS tools have been shown to reduce broad-spectrum antibiotic use by 13% and CLABSI rates by 33%.

ICU: intensive care unit; CDST: clinical decision support tool; BCC: blood culture contamination; CLABSI: central line associated bloodstream infection; SEP-1: severe sepsis and septic shock management bundle.

# References

1.	Wang MS, Zimmerman G, Klein T, et al. Three stages of laboratory stewardship in improving appropriate Clostridioides difficile testing in a community-based setting. *Antimicrob Steward Healthc Epidemiol*. 2025;5:e81.

2.	Planche T, Wilcox MH. Diagnostic pitfalls in Clostridium difficile infection. *Infect Dis Clin North Am*. Mar 2015;29:63-82.

3.	Liao JX, Appaneal HJ, Vicent ML, Vyas A, LaPlante KL. Path of least recurrence: A systematic review and meta-analysis of fidaxomicin versus vancomycin for Clostridioides difficile infection. *Pharmacotherapy*. Nov 2022;42:810-827.

4.	Rock C, Abosi O, Bleasdale S, et al. Clinical Decision Support Systems to Reduce Unnecessary Clostridioides difficile Testing Across Multiple Hospitals. *Clin Infect Dis*. Sep 30 2022;75:1187-1193.

5.	Khuvis J, Alsoubani M, Mae Rodday A, Doron S. The impact of diagnostic stewardship interventions on Clostridiodes difficile test ordering practices and results. *Clinical biochemistry*. Jul 2023;117:23-29.

6.	Bischoff W, Bubnov A, Palavecino E, et al. The Impact of Diagnostic Stewardship on Clostridium difficile Infections. *Open Forum Infectious Diseases*. 2017;4:S398.

7.	Polage CR, Gyorke CE, Kennedy MA, et al. Overdiagnosis of Clostridium difficile Infection in the Molecular Test Era. *JAMA Intern Med*. Nov 2015;175:1792-801.

8.	Guh AY, Fridkin S, Goodenough D, et al. Potential underreporting of treated patients using a Clostridioides difficile testing algorithm that screens with a nucleic acid amplification test. *Infect Control Hosp Epidemiol*. May 2024;45:590-598.

9.	Yogo A, Saade EA, Ransom EM, et al. Implementation of conditional reflex urine culturing decreases unnecessary antimicrobial use. *Infect Control Hosp Epidemiol*. Dec 2025;46:1228-1233.

10.	Yeates AC, Zimmerman JA, Destache CJ, Quimby DS. Cost analysis of urine testing for hospitalized patients in the emergency department. *Proc (Bayl Univ Med Cent)*. 2025;38:449-452.

11.	Webb BJ, Subramanian A, Lopansri B, et al. Antibiotic Exposure and Risk for Hospital-Associated Clostridioides difficile Infection. *Antimicrob Agents Chemother*. Mar 24 2020;64.

12.	Liu B, Reid J, Silverman M, Welk B. High risk of Clostridium difficile infection among spinal cord injured patients after the use of antibiotics commonly used to treat urinary tract infections. *Neurourol Urodyn*. Nov 2020;39:2401-2408.

13.	Agency for Healthcare Research and Quality. Estimating the Additional Hospital Inpatient Cost and Mortality Associated With Selected Hospital-Acquired Conditions. Accessed August 28, 2024, <https://www.ahrq.gov/hai/pfp/haccost2017-results.html>

14.	Claeys KC, Zhan M, Pineles L, et al. Conditional reflex to urine culture: Evaluation of a diagnostic stewardship intervention within the Veterans' Affairs and Centers for Disease Control and Prevention Practice-Based Research Network. *Infect Control Hosp Epidemiol*. Feb 2021;42:176-181.

15.	Claeys KC, Brown CH, Pineles L, et al. Implementation of Diagnostic Stewardship to Improve Urinary Tract Infection Antibiotic Use Across 3 Medical Centers. *Clin Infect Dis*. Mar 17 2026;82:494-500.

16.	Molloy MJ, Hall M, Markham JL, et al. Trends in Respiratory Pathogen Testing at US Children's Hospitals. *JAMA Netw Open*. Mar 3 2025;8:e250160.

17.	California Health and Human Services Agency. *Emergency department volume and capacity*. Department of Health Care Access and Information HAB; 2025. November 6, 2025. Accessed April 27, 2026. <https://data.chhs.ca.gov/dataset/emergency-department-volume-and-capacity>

18.	Centers for Medicare & Medicaid Services. Billing and Coding: Respiratory Pathogen Panel Testing. CMS. Accessed January 29, 2026. <https://www.cms.gov/medicare-coverage-database/view/article.aspx?articleid=58741&ver=17&utm>

19.	Ellis B, Chen K, Anicich K, et al. Optimization of the Extended Respiratory Panel Utilization via an Electronic Health Record Best Practice Alert. *J Mol Diagn*. May 2026;28:391-392.

20.	Kressel A, Cheatham M, Chang A. Diagnostic Stewardship of Respiratory Pathogen Panel Utilization. *Antimicrobial Stewardship & Healthcare Epidemiology*. 2021;1:s59.

21.	Fabre V, Hsu YJ, Carroll KC, et al. Multicenter evaluation of blood culture contamination and blood cultures practices in US acute care hospitals: time for standardization. *J Clin Microbiol*. Aug 13 2025;63:e0053025.

22.	Pliakos EE, Andreatos N, Shehadeh F, Ziakas PD, Mylonakis E. The Cost-Effectiveness of Rapid Diagnostic Testing for the Diagnosis of Bloodstream Infections with or without Antimicrobial Stewardship. *Clin Microbiol Rev*. Jul 2018;31.

23.	Woods-Hill CZ, Colantuoni EA, Koontz DW, et al. Association of Diagnostic Stewardship for Blood Cultures in Critically Ill Children With Culture Rates, Antibiotic Use, and Patient Outcomes: Results of the Bright STAR Collaborative. *JAMA Pediatr*. Jul 1 2022;176:690-698.

24.	Skoglund E, Dempsey CJ, Chen H, Garey KW. Estimated Clinical and Economic Impact through Use of a Novel Blood Collection Device To Reduce Blood Culture Contamination in the Emergency Department: a Cost-Benefit Analysis. *J Clin Microbiol*. Jan 2019;57.

25.	Fabre V, Klein E, Salinas AB, et al. A Diagnostic Stewardship Intervention To Improve Blood Culture Use among Adult Nonneutropenic Inpatients: the DISTRIBUTE Study. *J Clin Microbiol*. Sep 22 2020;58.

26.	Sautter RL, Parrott JS, Nachamkin I, et al. American Society for Microbiology evidence-based laboratory medicine practice guidelines to reduce blood culture contamination rates: a systematic review and meta-analysis. *Clin Microbiol Rev*. Dec 10 2024;37:e0008724.
